# A 15-Year Study on Up_4_A in Cardiovascular Disease

**DOI:** 10.3389/fphar.2020.01200

**Published:** 2020-08-04

**Authors:** Zhichao Zhou, Takayuki Matsumoto

**Affiliations:** ^1^ Division of Cardiology, Department of Medicine, Karolinska University Hospital, Karolinska Institutet, Stockholm, Sweden; ^2^ Department of Physiology and Morphology, Institute of Medicinal Chemistry, Hoshi University, Tokyo, Japan

**Keywords:** Up_4_A, cardiovascular, purinergic receptor, adenosine, ATP

## Introduction

In 2005, a novel dinucleotide uridine adenosine tetraphosphate (Up_4_A) was identified in the endothelium thereby being recognized as a novel endothelium-derived factor (Jankowski et al., 2005). Up_4_A is the first dinucleotide found in living organisms containing both a purine and a pyrimidine moieties (Jankowski et al., 2005). Up_4_A is biosynthesized through vascular endothelial growth factor receptor (VEGFR) 2 in response to pharmacological and mechanical stimuli (Jankowski et al., 2013). The plasma concentrations of Up_4_A in healthy subjects are in the vasoactive range suggesting that Up_4_A may contribute to cardiovascular regulation (Jankowski et al., 2005). Of importance, several pieces of evidence have reported that the plasma level of Up_4_A is elevated in patients with hypertension and with chronic kidney diseases, and that Up_4_A (via intra-aortic bolus injection) increases mean arterial pressure in rats *in vivo* (Jankowski et al., 2005; Jankowski et al., 2007; Schuchardt et al., 2012). These observations suggest a potential role for Up_4_A in the pathogenesis of cardiovascular disease.

During the following years, the research was focused on the vascular effect of Up_4_A in different vascular beds of various species. This mainly includes the acute effect of Up_4_A on vascular function in both health and various cardiovascular diseases including hypertension, atherosclerosis, myocardial infarction, and diabetes, and the trophic effect of Up_4_A on vascular proliferation, migration, angiogenesis, and calcification (Matsumoto et al., 2015; Zhou et al., 2019). Up_4_A exerts biological effects by activating purinergic receptors (PRs) to regulate cardiovascular (dys)function. PRs are divided into P1R and P2R categories. Four subtypes of P1Rs (adenosine receptors) have been identified, namely A1R, A_2A_R, A_2B_R, and A3R. At least seven P2XRs, and eight P2YRs have been identified to date (Burnstock, 2017). In the vasculature, activation of A1R and A3R can induce contraction, whereas activation of A_2A_R and A_2B_R typically produce vascular relaxation (Zhou et al., 2019). In contrast to P1Rs, the effects of the activation of P2R subtypes may be tissue- and cell-dependent. In general, activation of P2R subtypes in endothelial cells are thought to induce vasodilation, while activation of P2Rs in smooth muscle cells can induce vasoconstriction (Zhou et al., 2019). Of note, the pharmacological action of Up_4_A on vascular function and the Up_4_A-mediated purinergic signaling have been shown to be altered in cardiovascular disease (Zhou et al., 2019). However, the endogenous role of Up_4_A in the regulation of cardiovascular homeostasis and particularly the role of Up_4_A in the development and progression of cardiovascular disease remain largely unclear. This study briefly summarizes the available information regarding the vascular action of Up_4_A and its mediated purinergic signaling in various cardiovascular diseases during a 15-year research period and raises critical questions and perspectives for the future research direction in order to better understand the biological role of Up_4_A in the development of cardiovascular disease.

## Up_4_A Biosynthesis and Catabolism

Up_4_A is biosynthesized by activation of VEGFR2 in the endothelium (Jankowski et al., 2013). After incubation of ADP and UDP with VEGFR2, human dermal endothelial cells generate increasing concentrations of Up_4_A, while there is no Up_4_A formation when incubating ADP and UDP with VEGFR1 or VEGFR3 (Jankowski et al., 2013). The domain of Tyr-1175 of VEGFR2 is essential for the enzymatic activity for Up_4_A synthesis (Jankowski et al., 2013). Up_4_A could facilitate VEGFR2-mediated signaling pathways e.g. p42/44 mitogen-activated protein kinase phosphorylation (Jankowski et al., 2013). Given that VEGFR2 is abundantly expressed in endothelial cells, the Up_4_A biosynthesis may play a significant role in cardiovascular homeostasis.

In addition to endothelial cells, Up_4_A was found to be generated in renal tubular cells, human liver hepatocellular carcinoma cells, human acute monocytic leukemia cells, and murine macrophage cells (Zhou et al., 2019). As these cells express VEGFR2, Up_4_A synthesis is likely also mediated by activation of VEGFR2. It is of interest to know whether VEGFR2-expressing cells are generally capable of synthesizing Up_4_A. Of further importance, several cardiovascular diseases including diabetes have demonstrated an altered VEGFR2 expression and function (Fountas et al., 2015). Whether such alteration may affect Up_4_A generation and subsequent purinergic activation accounting for the development and progression of disease remains unknown and warrants further studies.

Catabolism of Up_4_A is poorly understood. Dinucleotides can be degraded to mononucleotides by ecto-nucleotidases (including CD39 and CD37) (Burnstock, 2017). These ecto-nucleotidases are ubiquitously present in different cells, including vascular endothelial and smooth muscle cells (Burnstock, 2017). This implies that Up_4_A may be also degraded through those ecto-nucleotidases, and that the Up_4_A-mediated vascular effects can be exerted by its degradation products. However, existing evidence suggest that Up_4_A-mediated vascular effects are direct, but not indirect through its degradation or inhibition of purinergic enzymes as mentioned above (Zhou et al., 2019). It is speculated that the catabolism of Up_4_A may be through other types of ecto-nucleotidases e.g. nucleotide pyrophosphatase/phosphodiesterases. Further studies are needed to explore this mechanism. Of note, activity of these nucleotidases are altered in cardiovascular disease (Burnstock, 2017; Zhou et al., 2020). It is of importance to know in the future studies whether altered nucleotidase activity may influence the catabolism of Up_4_A contributing to the initiation and/or development of cardiovascular disease.

## Up_4_A and Vascular Actions

Using pharmacological approach, the 15-year research on Up_4_A has unveiled vascular effects of Up_4_A in various cardiovascular diseases. The Up_4_A-mediated PR activation and possible downstream pathways have been characterized in hypertension, diabetes, atherosclerosis, and myocardial infarction. In addition, Up_4_A exerts various trophic effects through activation of P2YRs inducing angiogenesis in endothelial cells, proliferation and migration in smooth muscle cells, and development of calcification (Zhou et al., 2019). These chronic effects may also play a role for vascular remodeling and atherogenesis.

### Hypertension

Vascular reactivity to Up_4_A is altered in hypertension. In deoxycorticosterone acetate-salt rats, Up_4_A-induced contraction is heterogeneously affected among various vessels. Thus, Up_4_A-induced vascular contraction was increased in basilar, renal, and femoral arteries, but was decreased in small mesenteric arteries, and unchanged in thoracic aortas and pulmonary arteries (Matsumoto et al., 2011; Matsumoto et al., 2012). Activation of P2YR contributed to the increased Up_4_A-induced contraction in basilar, femoral, and renal arteries (Matsumoto et al., 2011; Matsumoto et al., 2012). In angiotensin II-induced hypertensive mice, Up_4_A-induced contraction in aortas was decreased likely due to P2X_1_R desensitization (Zhou et al., 2017b). These observations may imply that vascular PR activity rather than circulating Up_4_A may determine the role of Up_4_A in setting of hypertension. However, further investigations regarding the relationship between altered circulating Up_4_A and vascular PR activity in hypertension are needed. In contrast to vasoconstrictor effect, Up_4_A produces potent relaxation in porcine coronary small arteries (Zhou et al., 2013b; Sun et al., 2019). In a swine model with pressure-overload-induced hypertension, Up_4_A-induced relaxation was impaired in coronary small arteries isolated from hypertensive swine compared to control. ARs other than A_2A_R and P2Y_12_R among P2Rs are proposed contributing to the reduced relaxation to Up_4_A in hypertensive swine (Zhou et al., 2018a).

### Diabetes

Diabetes is an important risk factor for the development of cardiovascular diseases including atherosclerosis and ischemic heart disease (Pereira et al., 2018; Zhou et al., 2018b). Diabetes-associated vascular complications are the leading causes of increased morbidity and mortality worldwide (Zhou et al., 2018b). Up_4_A-induced contraction in renal arteries of healthy rats, which was enhanced in vessels from Goto-Kakizaki (GK) rats likely due to activation of suramin-sensitive P2Rs (Matsumoto et al., 2014). Up_4_A-induced contraction was decreased in aortas and renal arteries of the Otsuka Long-Evans Tokushima Fatty (OLETF) diabetic rats as compared to control Long-Evans Tokushima Otsuka (LETO) rats at basal tone (Matsumoto et al., 2016; Matsumoto et al., 2017). Of note, the Up_4_A-induced renal contraction in OLETF rats was increased with age and duration of diabetes, whereas the Up_4_A-induced contraction in LETO rats was not associated with age (Matsumoto et al., 2016). With elevated tone by phenylephrine, Up_4_A produced a mild relaxation in aortas isolated from OLETF rats as compared to the vasoconstrictor effect by Up_4_A in LETO rats (Matsumoto et al., 2017). The involvements of PRs in different vascular responses to Up_4_A in this model need further investigations. However, Up_4_A can stimulate other endothelium-derived factors e.g. vasoconstrictor prostanoids PGF_2α_, PGE_2_, and thromboxane (TxA_2_) are generated in response to Up_4_A to promote its contraction and nitric oxide is released to suppress Up_4_A effects in OLETF rats (Matsumoto et al., 2017). Interestingly, Up_4_A-induced relaxation in coronary small arteries was maintained in swine with diabetes and endothelial dysfunction compared to normal swine (Zhou et al., 2017a). This is due to a balanced purinergic activation (reduced vasodilator A_2A_R and P2X_7_R vs. increased vasodilator P2Y_1_R) and endothelium-derived factor-mediated effects (vasodilator CYP 2C9 vs. vasoconstrictor CYP 2C9 and TxA2) in response to Up_4_A (Zhou et al., 2017a).

### Coronary Atherosclerosis and Myocardial Infarction

Plaque formation due to atherosclerosis in coronary vasculature is a major cause of ischemic heart disease. When the plaque ruptures, the ensuing thromboembolism may lead to ischemia and myocardial infarction (Marzilli et al., 2012). Despite all four ARs and many P2Rs are involved in the development of atherosclerosis and targeting P2Y_12_R is an effective strategy commonly used in patients with acute coronary syndrome (Burnstock, 2017), the experimental evidence for the involvement of PRs in coronary atherosclerosis is lacking. In coronary arteries isolated from *ApoE* knockout mice treated with a high fat diet, in which lesions were observed, P2X_1_R expression was decreased in endothelial cells, while P2X_1_R expression remained unaltered in smooth muscle cells. Hence, the smooth muscle to endothelial cell ratio of P2X_1_R was increased, suggesting a net vasoconstrictor effect of P2X_1_R in coronary atherosclerosis (Teng et al., 2017). Indeed, infusion of Up_4_A into isolated hearts from *ApoE* knockout mice with high fat diet decreased coronary flow more as compared to hearts from control mice through activation of vasoconstrictor P2X_1_R (Teng et al., 2017). In contrast to *ex vivo* experiments, a bolus *i.v.* injection of Up_4_A increased coronary blood flow to a similar extent between control and atherosclerotic mice (Teng et al., 2017). This vasodilator effect of Up_4_A is not influenced by the hemodynamic changes by the drug infusion. However, the possibility of a Up_4_A degradation to purine or adenosine to induce coronary vasodilation *in vivo* condition remain undetermined, which warrants further investigations. In swine after myocardial infarction, the sensitivity of the coronary small arteries to Up_4_A was reduced (Zhou et al., 2013a). The reduced vasodilator response to Up_4_A is due to a reduced contribution of P1R (A_2B_R was proposed to be involved) (Zhou et al., 2013a).

## Conclusions and Perspective

The 15-year research on Up_4_A in cardiovascular system has yielded fruitful outcomes. Up_4_A produces both short-term and long-term vascular effects through both P1R and P2R. The Up_4_A-induced effects not only depend on various vascular beds but also different species. The involvement of PRs in response to Up_4_A is altered in various cardiovascular diseases. However, endogenous role of Up_4_A in the regulation of vascular function remains unclear. Although the vasoactive effect of Up_4_A may depend on receptor activity, the contribution of the Up_4_A plasma levels to vascular (dys)function in cardiovascular disease warrants further investigations. Future research directions need to focus on the following aspects to better understand the role of Up_4_A in the development and progression of cardiovascular disease: 1) Does the altered plasma levels of Up_4_A in cardiovascular disease merely serve as a diagnostic biomarker, and/or can endogenous Up_4_A (including local concentration of Up_4_A e.g. in coronary microcirculation) activate corresponding PRs serving as a causative factor? What are causal factors and mechanisms underlying regulation of local/circulating Up_4_A levels? 2) Given that Up_4_A can activate both P1Rs and P2Rs expressed in different cells of the cardiovascular system, it remains to be investigated which receptors play an essential role. 3) Can Up_4_A biosynthesis and its mediated main purinergic signaling be targeted for the treatment of cardiovascular disease? Whether targeting a single receptor or multiple receptors at the same time yields in the most effective therapeutic effects. 4) What are the mechanisms underlying the Up_4_A-mediated effect *in vivo* condition? 5) Can Up_4_A-mediated vascular effect be eventually translated into human situation? It may take many other 15 years to address all these important questions and concerns. However, the successful characterization of vascular effect of Up_4_A and PRs involved in cardiovascular disease mentioned in the present study have paved the way for the next research step.

## Dedication

This study is dedicated to the memory of Geoffrey Burnstock from Royal Free and University College Medical School, London, United Kingdom, who peacefully passed away on June 3^rd^ 2020.

## Author Contributions

ZZ conceived the study. ZZ and TM wrote and edited the manuscript. All authors contributed to the article and approved the submitted version.

## Funding

This work was supported by the Swedish Heart and Lung Foundation (20190341 to ZZ), the Loo and Hans Ostermans Foundation (2018-01213 and 2020-01209 to ZZ), and the Lars Hiertas Minne Foundation (FO2018-0156 to ZZ).

## Conflict of Interest

The authors declare that the research was conducted in the absence of any commercial or financial relationships that could be construed as a potential conflict of interest.

## References

[B1] Burnstock G. (2017). Purinergic Signaling in the Cardiovascular System. Circ. Res. 120 (1), 207–228. 10.1161/CIRCRESAHA.116.309726 28057794

[B2] Fountas A. Diamantopoulos L. N. Tsatsoulis A. (2015). Tyrosine Kinase Inhibitors and Diabetes: A Novel Treatment Paradigm? Trends Endocrinol. Metab. 26 (11), 643–656. 10.1016/j.tem.2015.09.003 26492832

[B3] Jankowski V. Tolle M. Vanholder R. Schonfelder G. van der Giet M. Henning L. (2005). Uridine adenosine tetraphosphate: a novel endothelium- derived vasoconstrictive factor. Nat. Med. 11 (2), 223–227. 10.1038/nm1188 15665829

[B4] Jankowski V. Meyer A. A. Schlattmann P. Gui Y. Zheng X. L. Stamcou I. (2007). Increased uridine adenosine tetraphosphate concentrations in plasma of juvenile hypertensives. Arterioscler. Thromb. Vasc. Biol. 27 (8), 1776–1781. 10.1161/ATVBAHA.107.143958 17569882

[B5] Jankowski V. Schulz A. Kretschmer A. Mischak H. Boehringer F. van der Giet M. (2013). The enzymatic activity of the VEGFR2 receptor for the biosynthesis of dinucleoside polyphosphates. J. Mol. Med. (Berl) 91 (9), 1095–1107. 10.1007/s00109-013-1036-y 23636508

[B6] Marzilli M. Merz C. N. Boden W. E. Bonow R. O. Capozza P. G. Chilian W. M. (2012). Obstructive coronary atherosclerosis and ischemic heart disease: an elusive link! J. Am. Coll. Cardiol. 60 (11), 951–956. 10.1016/j.jacc.2012.02.082 22954239

[B7] Matsumoto T. Tostes R. C. Webb R. C. (2011). Uridine adenosine tetraphosphate-induced contraction is increased in renal but not pulmonary arteries from DOCA-salt hypertensive rats. Am. J. Physiol. Heart Circ. Physiol. 301 (2), H409–H417. 10.1152/ajpheart.00084.2011 21551273 PMC3154666

[B8] Matsumoto T. Tostes R. C. Webb R. C. (2012). Alterations in vasoconstrictor responses to the endothelium-derived contracting factor uridine adenosine tetraphosphate are region specific in DOCA-salt hypertensive rats. Pharmacol. Res. 65 (1), 81–90. 10.1016/j.phrs.2011.09.005 21933714 PMC3264768

[B9] Matsumoto T. Watanabe S. Kawamura R. Taguchi K. Kobayashi T. (2014). Enhanced uridine adenosine tetraphosphate-induced contraction in renal artery from type 2 diabetic Goto-Kakizaki rats due to activated cyclooxygenase/thromboxane receptor axis. Pflugers Arch. 466 (2), 331–342. 10.1007/s00424-013-1330-0 23900807

[B10] Matsumoto T. Goulopoulou S. Taguchi K. Tostes R. C. Kobayashi T. (2015). Constrictor prostanoids and uridine adenosine tetraphosphate: vascular mediators and therapeutic targets in hypertension and diabetes. Br. J. Pharmacol. 172 (16), 3980–4001. 10.1111/bph.13205 26031319 PMC4543607

[B11] Matsumoto T. Watanabe S. Ando M. Yamada K. Iguchi M. Taguchi K. (2016). Diabetes and Age-Related Differences in Vascular Function of Renal Artery: Possible Involvement of Endoplasmic Reticulum Stress. Rejuvenation Res. 19 (1), 41–52. 10.1089/rej.2015.1662 26234558

[B12] Matsumoto T. Kobayashi S. Ando M. Iguchi M. Takayanagi K. Kojima M. (2017). Alteration of Vascular Responsiveness to Uridine Adenosine Tetraphosphate in Aortas Isolated from Male Diabetic Otsuka Long-Evans Tokushima Fatty Rats: The Involvement of Prostanoids. Int. J. Mol. Sci. 18 (11), 2378. 10.3390/ijms18112378 PMC5713347 29120387

[B13] Pereira C. A. Carneiro F. S. Matsumoto T. Tostes R. C. (2018). Bonus Effects of Antidiabetic Drugs: Possible Beneficial Effects on Endothelial Dysfunction, Vascular Inflammation and Atherosclerosis. Basic Clin. Pharmacol. Toxicol. 123 (5), 523–538. 10.1111/bcpt.13054 29890033

[B14] Schuchardt M. Tolle M. Prufer J. Prufer N. Huang T. Jankowski V. (2012). Uridine adenosine tetraphosphate activation of the purinergic receptor P2Y enhances in vitro vascular calcification. Kidney Int. 81 (3), 256–265. 10.1038/ki.2011.326 21956191

[B15] Sun C. Jiao T. Merkus D. Duncker D. J. Mustafa S. J. Zhou Z. (2019). Activation of adenosine A2A but not A2B receptors is involved in uridine adenosine tetraphosphate-induced porcine coronary smooth muscle relaxation. J. Pharmacol. Sci. 141 (1), 64–69. 10.1016/j.jphs.2019.09.006 31640919 PMC7418061

[B16] Teng B. Labazi H. Sun C. Yang Y. Zeng X. Mustafa S. J. (2017). Divergent coronary flow responses to uridine adenosine tetraphosphate in atherosclerotic ApoE knockout mice. Purinergic Signal 13 (4), 591–600. 10.1007/s11302-017-9586-z 28929376 PMC5714849

[B17] Zhou Z. de Wijs-Meijler D. Lankhuizen I. Jankowski J. Jankowski V. Jan Danser A. H. (2013a). Blunted coronary vasodilator response to uridine adenosine tetraphosphate in post-infarct remodeled myocardium is due to reduced P1 receptor activation. Pharmacol. Res. 77, 22–29. 10.1016/j.phrs.2013.08.007 23994209

[B18] Zhou Z. Merkus D. Cheng C. Duckers H. J. Jan Danser A. H. Duncker D. J. (2013b). Uridine adenosine tetraphosphate is a novel vasodilator in the coronary microcirculation which acts through purinergic P1 but not P2 receptors. Pharmacol. Res. 67 (1), 10–17. 10.1016/j.phrs.2012.09.011 23063485

[B19] Zhou Z. Sorop O. de Beer V. J. Heinonen I. Cheng C. Jan Danser A. H. (2017a). Altered purinergic signaling in uridine adenosine tetraphosphate-induced coronary relaxation in swine with metabolic derangement. Purinergic Signal 13 (3), 319–329. 10.1007/s11302-017-9563-6 28540569 PMC5563292

[B20] Zhou Z. Yadav V. R. Sun C. Teng B. Mustafa J. S. (2017b). Impaired Aortic Contractility to Uridine Adenosine Tetraphosphate in Angiotensin II-Induced Hypertensive Mice: Receptor Desensitization? Am. J. Hypertens. 30 (3), 304–312. 10.1093/ajh/hpw163 28034895 PMC5861566

[B21] Zhou Z. Lankhuizen I. M. van Beusekom H. M. Cheng C. Duncker D. J. Merkus D. (2018a). Uridine Adenosine Tetraphosphate-Induced Coronary Relaxation Is Blunted in Swine With Pressure Overload: A Role for Vasoconstrictor Prostanoids. Front. Pharmacol. 9, 255. 10.3389/fphar.2018.00255 29632487 PMC5879110

[B22] Zhou Z. Mahdi A. Tratsiakovich Y. Zahoran S. Kovamees O. Nordin F. (2018b). Erythrocytes From Patients With Type 2 Diabetes Induce Endothelial Dysfunction Via Arginase I. J. Am. Coll. Cardiol. 72 (7), 769–780. 10.1016/j.jacc.2018.05.052 30092954

[B23] Zhou Z. Matsumoto T. Jankowski V. Pernow J. Mustafa S. J. Duncker D. J. (2019). Uridine adenosine tetraphosphate and purinergic signaling in cardiovascular system: An update. Pharmacol. Res. 141, 32–45. 10.1016/j.phrs.2018.12.009 30553823 PMC6685433

[B24] Zhou R. Dang X. Sprague R. S. Mustafa S. J. Zhou Z. (2020). Alteration of purinergic signaling in diabetes: Focus on vascular function. J. Mol. Cell Cardiol. 140, 1–9. 10.1016/j.yjmcc.2020.02.004 32057736 PMC7425578

